# Treatment of corneal neovascularization in ocular chemical injury with an off-label use of subconjunctival bevacizumab: a case report

**DOI:** 10.1186/1752-1947-7-199

**Published:** 2013-07-26

**Authors:** Ludovico Iannetti, Alessandro Abbouda, Claudia Fabiani, Roberta Zito, Michelangelo Campanella

**Affiliations:** 1 Department of Ophthalmology, University of Rome “La Sapienza,” , Rome, Italy; 2 Department of Comparative Biomedical Sciences, The Royal Veterinary College and UCL Consortium for Mitochondrial Research, University of London, London, UK; 3 European Brain Research Institute (EBRI), Rita Levi-Montalcini Foundation, Rome, Italy; 4 Ocular Immunovirology Service, University of Rome “La Sapienza,” , Rome, Italy

**Keywords:** Chemical injuries, Corneal neovascularization, Bevacizumab, Conjunctival injection

## Abstract

**Introduction:**

In this report, we describe the case of a patient with ocular chemical injury, symblepharon, and corneal neovascularization in whom subconjunctival injection of bevacizumab caused regression of corneal opacification and neovascularization, which led to visual improvement.

**Case presentation:**

A 54-year-old Caucasian woman presented at our eye emergency department following a splash injury of the left eye with sodium hydroxide. At presentation, her visual acuity was light perception. Slit-lamp examination showed diffuse corneal epithelial defects, stromal edema, and localized Descemet’s folds. Despite administration of topical and systemic steroids, she developed symblepharon after 3 months as well as superficial and deep corneal neovascularization with visual acuity 0.5 logarithm of the minimum angle of resolution. A subconjunctival bevacizumab injection (dose 1.25mg/0.05ml) was administered. After 1 week, the vessels appeared thinner and corneal opacity was clearer. Her visual acuity improved to 0.3 logarithm of the minimum angle of resolution. Three weeks later her visual acuity had not changed, and the vessels had started to perfuse again. A second subconjunctival bevacizumab injection was given. After 2 weeks, her vision had improved to 0.1 logarithm of the minimum angle of resolution, vessel regression was observed, and corneal opacity was significantly reduced. Three months after the second injection her vision was unchanged, and the neovascularization remained stable. During the next months, the patient’s condition was well-controlled, and, at the end of follow-up 24 months later, her visual acuity and clinical condition were unaltered.

**Conclusion:**

Subconjunctival bevacizumab injection may be considered as a second-line treatment of corneal neovascularization caused by chemical injury that is unresponsive to conventional steroid therapy.

## Introduction

Ocular chemical and thermal burns are a devastating cause of corneal blindness characterized by limbal stem cell deficiency, stromal opacification, ocular surface keratinization, corneal and/or conjunctival or ocular surface neovascularization, and high corneal graft failure rates. Persistent corneal epithelial defects may lead to tissue thinning or melting, perforation, and secondary infections. The deepithelialized conjunctival surfaces tend to fuse and form symblepharon bands. The severity of the injury depends on the type of offending agent, its concentration, the duration of exposure, and the extent of contact [1]. Conventional medical therapies include steroids, ascorbate, citrates, tetracyclines, lubricants, and surgical procedures such as the application of a contact lens and amniotic membrane transplantation [2]. Recently, the use of subconjunctival bevacizumab, a humanized monoclonal antibody targeting vascular endothelial growth factor (VEGF), has been proposed [3-5]. A recent case report described the use of bevacizumab in a child with Stevens-Johnson syndrome [3].

We report the case of a patient with ocular chemical injury, symblepharon, and corneal neovascularization in whom subconjunctival injection of bevacizumab caused regression of corneal opacification and neovascularization with resulting visual improvement.

## Case presentation

In this report, we describe a retrospective, interventional case of a 54-year-old Caucasian woman who presented at our eye emergency department following a splash injury of the left eye with sodium hydroxide. This report was reviewed and approved by the ethics committee of the University of Rome “La Sapienza.”

**Figure 1:**
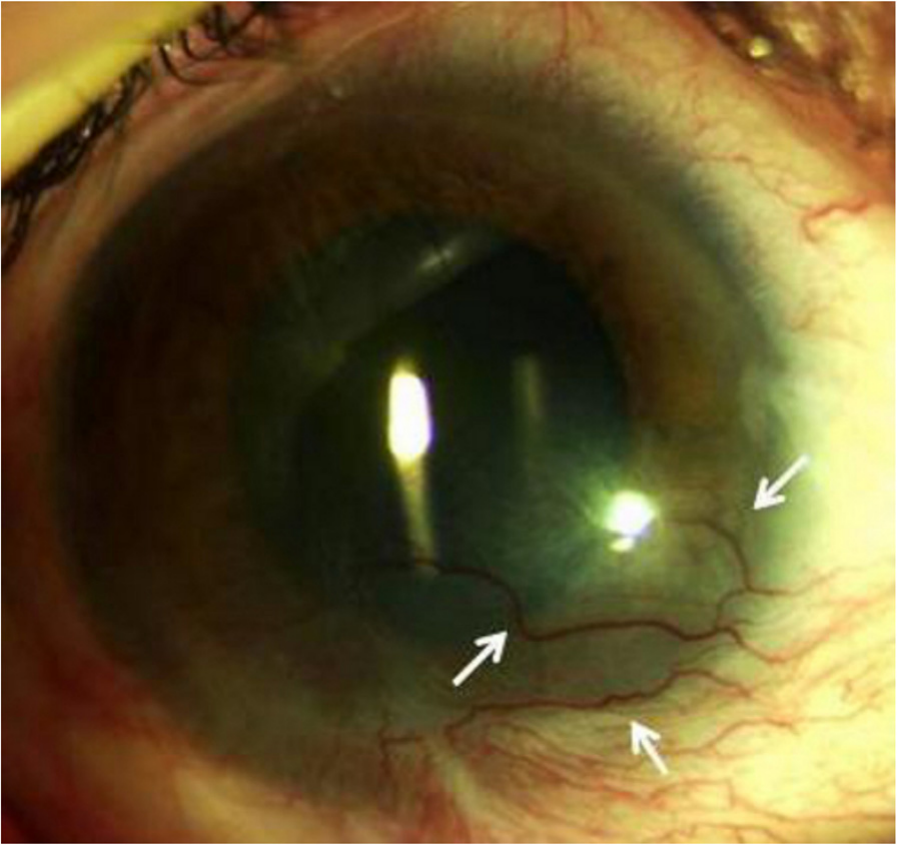
Pre-injection examination revealed symblepharon and corneal opacity in the inferior sector with superficial and deep corneal neovascularization (arrows).

**Figure 2:**
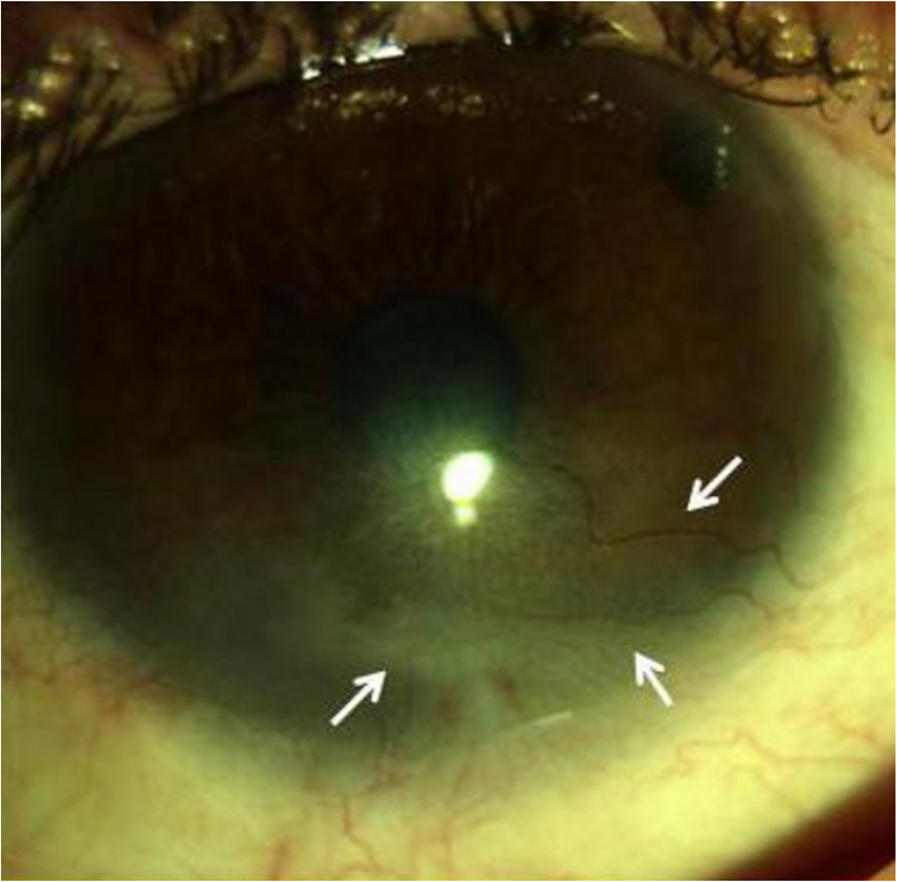
Examination 3 months after the second injection of bevacizumab (dose 1.25mg/0.05ml) revealed inferior scarring and significant reduction of corneal new vessels in number and size (arrows).

At the first examination, her visual acuity (VA) was light perception in the left eye. Her eyelids and conjunctiva were congested. A slit-lamp examination showed a diffuse corneal epithelial defect, stromal edema, and localized Descemet’s folds. She was treated with 1% dexamethasone sodium phosphate drops six times daily, 1% atropine drops three times daily, and tobramycin six times daily. The corticosteroid prednisone 50mg was administered daily for the first 10 days, then the daily dose was gradually tapered by 5mg every 15 days. Three months later, despite the frequent use of topical steroids a symblepharon with superficial and deep corneal neovascularization was observed (Figure 1). The patient’s VA was 0.5 logarithm of the minimum angle of resolution (LogMAR). We suggested administering a subconjunctival bevacizumab injection in the left eye with the aim of reducing corneal neovascularization. Written, informed consent was obtained from the patient after explaining the off-label use of bevacizumab on the basis of two case reports [3,4]. A subconjunctival injection of 0.05ml (1.25mg) of sterile, undiluted, commercially available bevacizumab (Avastin®; Genentech, South San Francisco, CA, USA) was administered with topical anesthesia in the subconjunctival space close to the corneal limbus and adjacent to the pathological blood vessels. After the procedure, the patient was advised to use 0.3% ofloxacin and 1% dexamethasone sodium phosphate drops three times daily for one week. One week after the subconjunctival bevacizumab injection, the vessels appeared thinner and the corneal opacity was clearer. The patient’s VA improved to 0.3LogMAR after one week. Three weeks later her VA had not changed, and the vessels started to perfuse again. A second subconjunctival bevacizumab injection was then given. Two weeks after the second injection her VA improved to 0.1LogMAR and corneal neovascularization was significantly reduced. Notably, three months later her vision was unchanged and the neovascularization remained stable (Figure 2). During the next months, the patient’s condition was well-controlled, and, at the end of follow-up 24 months later, her VA and clinical condition were unaltered.

## Discussion

The off-label use of bevacizumab in patients with ophthalmic diseases involving neovascularization as the primary or secondary pathology has improved the management of several diseases, such as age-related macular degeneration, diabetic retinopathy, corneal neovascularization, neovascular glaucoma, and retinopathy of prematurity. Ocular surface neovascularization is a prominent feature of ocular chemical injury that causes visual loss as a result of associated scarring and lipid deposition. Conventional medical therapies include steroids, ascorbate, citrates, tetracyclines, lubricants, and surgical procedures such as application of a contact lens and amniotic membrane transplantation [2]. Our data indicate that, when conventional approaches fail, treatment with subconjunctival bevacizumab may be effective [3-5]. By giving two subconjunctival bevacizumab injections, we obtained a dramatic improvement of VA, and this effect was magnified by the anti-VEGF treatment that led to a significant reduction of corneal neovascularization. A causal role of the VEGF in promoting ocular surface neovascularization was thus confirmed [6].

## Conclusion

To the best of our knowledge, this is one of the first case reports describing the long-term results of subconjunctival bevacizumab treatment in corneal neovascularization secondary to ocular chemical injury. Subconjunctival bevacizumab injection may be considered as an optional treatment in patients who do not respond to conventional steroid therapy. Future studies will explore this therapy toward a feasible standardization.

## Consent

Written informed consent was obtained from the patient for publication of this case report and any accompanying images. A copy of the written consent is available for review by the Editor-in-Chief of this journal.

## Competing interests

The authors declare that they have no competing interests.

## Authors’ contribution

LI performed the procedure of treatment and was the principal investigator of the study. AA contributed to image production. CF and RZ collected the clinical data. MC gave precious and valuable contributions in writing and revising the manuscript. All authors read and approved the final manuscript.
